# Individual dynamics of daily life functioning of reward system can predict future level of depressive symptoms

**DOI:** 10.1192/j.eurpsy.2021.309

**Published:** 2021-08-13

**Authors:** A. Kuranova, A. Martinez, M. Wichers, J. Wigman, S. Booij

**Affiliations:** 1 Department Of Psychiatry, Interdisciplinary Center Psychopathology And Emotion Regulation (icpe), University of Groningen, University Medical Center Groningen, Groningen, Netherlands; 2 Faculty Of Psychology And Neuroscience, Maastricht University, Maastricht, Netherlands

**Keywords:** reward system, reward dynamics, Depression, Network analysis

## Abstract

**Introduction:**

The reward system regulates the processes that motivate people to pursue evolutionary beneficial stimuli. Effective functioning of the reward system can protect against the development of anhedonia. In the daily life, the reward system can be expressed as the dynamic interplay of positive affect (liking), reward anticipation (wanting), and active behavior (engaging). Applying network analysis to daily life experience data allows us to identify such reward dynamics and use them to predict future depressive symptoms.

**Objectives:**

We investigated whether at baseline (i) higher network positive affect in-strength, reflecting how strongly positive affect is influenced by other components and hence the level of anhedonia, and (ii) higher network connectivity, reflecting overall functioning of the reward system, are associated with fewer depressive symptoms on follow-up.

**Methods:**

We used data from 43 participants with mild depressive symptoms from the SMARTSCAN study. The dynamic interplay between momentary positive affect, reward anticipation, and active behavior was assessed with individual vector-autoregressive models and the network analysis. Network positive affect in-strength and connectivity indices were used to predict a six-month depressive symptoms trajectory.

**Results:**

Reward systems networks vary greatly between individuals. On the group level, higher positive affect in-strength (Beta=-3.66, p=0.05) and network connectivity (Beta=-4.06, p=0.03) at baseline were associated with fewer symptoms at follow-up.

**Conclusions:**

Higher influences of reward anticipation and active behavior on positive affect and stronger connections between reward cycle components are associated with fewer future symptoms, suggesting the importance of daily life reward cycle dynamics in depression.

**Disclosure:**

No significant relationships.

